# Mitochondrial Quality Control Imbalance in the Heterogeneity of Parkinson’s Disease: From Selective Vulnerability to Stratified Transformation

**DOI:** 10.3390/ijms27188086

**Published:** 2026-09-11

**Authors:** Yongxu Chen, Chunsheng Wang

**Affiliations:** College of Life Science, Northeast Forestry University, Harbin 150040, China; 2023214727@nefu.edu.cn

**Keywords:** Parkinson’s disease, mitochondrial quality control, selective vulnerability, heterogeneity, stratified transformation

## Abstract

Parkinson’s disease (PD) is a clinically and biologically heterogeneous neurodegenerative disorder in which variable symptom profiles, progression rates, and treatment responses likely reflect distinct but partially convergent pathogenic mechanisms. Among these, mitochondrial dysfunction recurs across both familial and sporadic PD; however, this broad concept alone cannot explain disease heterogeneity. To preserve mitochondrial homeostasis, cells rely on a complex mitochondrial quality control (MQC) system that encompasses protein import and proteostasis, redox surveillance, organellar dynamics and positioning, biogenesis, and selective elimination of damaged mitochondria. MQC also depends on coordination with other organelles, particularly the endoplasmic reticulum and lysosomes. In this review, we discuss how different layers of MQC maintain mitochondrial integrity and how these pathways are functionally coupled. We further consider how an MQC-based framework may help explain the clinical heterogeneity of PD, including selective neuronal vulnerability, subtype formation, and divergent disease progression, and how it can frame recent therapeutic advances aimed at biologically stratified intervention.

## 1. Introduction

Parkinson’s disease (PD) is increasingly recognized not as a single, uniform disorder, but as a biologically and clinically heterogeneous disease [1]. Although traditionally defined by its motor syndrome, PD varies markedly in prodromal manifestations, symptom profile, progression rate, treatment response, and prognosis [1,2,3,4]. These observations suggest that PD heterogeneity arises from underlying biological diversity rather than from superficial clinical variation.

Genetic and genomic studies indicate that PD risk is distributed across multiple pathogenic pathways, including lysosomal–autophagic dysfunction, impaired proteostasis, vesicular and endolysosomal trafficking defects, mitochondrial disturbance, and immune–inflammatory dysregulation [5,6,7]. In both familial PD (fPD) and sporadic PD (sPD), distinct etiological routes may converge on shared downstream stress states rather than entirely separate pathogenic endpoints. Among these, mitochondrial dysfunction is especially notable, because it recurs across heterogeneous forms of PD and may represent a key point of convergence [8]. Single-neuron analyses of substantia nigra pars compacta (SNpc) dopaminergic neurons in sPD have revealed broad oxidative phosphorylation defects across all five respiratory-chain complexes and reduced expression of multiple MQC-related proteins, despite preserved mitochondrial mass [9]. These findings suggest that mitochondrial impairment in PD reflects a disease-relevant disturbance in mitochondrial maintenance and adaptation.

However, mitochondrial dysfunction alone is too broad a concept to explain PD heterogeneity. Mitochondrial quality control (MQC) offers a more precise organizing framework, because it spans multiple, functionally integrated levels of mitochondrial maintenance [10]. Diverse upstream insults, including α-synuclein pathology, lysosomal dysfunction, PD-linked genetic defects, and inflammatory stress, may disrupt different MQC modules, generating distinct patterns of mitochondrial failure [11]. A central question, therefore, is how patient-specific differences in MQC dysfunction shape selective vulnerability, subtype formation, and divergent disease progression. Beyond its mechanistic importance, MQC is also relevant to clinical translation: because disease-modifying therapies remain limited, it may provide a framework for biologically stratified intervention by linking patient-specific patterns of mitochondrial, lysosomal, and trafficking dysfunction to targeted therapeutic strategies. This perspective may help guide biomarker development, patient selection, and mechanism-based trial design. This review therefore examines MQC as a mechanistic bridge between molecular heterogeneity and clinical heterogeneity in PD.

## 2. Multilevel Architecture of Mitochondrial Quality Control in PD

Under physiological conditions, neuronal mitochondrial quality control (MQC) is a graded homeostatic system rather than a synonym for mitophagy. It continuously detects mitochondrial stress, preserves components that remain functional, repairs or compensates for reversible defects, and removes organelles that can no longer be restored. These activities are particularly important in neurons because mitochondrial populations must supply ATP and buffer calcium at spatially separated sites, including long axons and presynaptic terminals, while adapting to changing local demand [12,13,14,15,16].

MQC can be considered at three functionally connected levels. At the molecular level, protein import, chaperones, proteases, redox buffering, mitochondrial DNA maintenance, and biogenesis preserve the mitochondrial proteome and respiratory competence. At the organelle level, fusion enables content mixing and partial complementation, whereas fission, transport arrest, and spatial redistribution help segregate damaged mitochondrial domains and position functional mitochondria where they are needed. When damage exceeds the capacity for repair or network-level compensation, selective mitochondrial elimination engages autophagic and lysosomal pathways to remove persistently dysfunctional organelles [12,13,14,15,16,17,18,19,20].

In the best-characterized damage-sensing pathway, loss of membrane potential or impaired protein import stabilizes PINK1 on the outer mitochondrial membrane. PINK1 phosphorylates pre-existing ubiquitin and the ubiquitin-like domain of Parkin at Ser65. Phosphorylated ubiquitin promotes Parkin recruitment and primes it for PINK1-dependent activation, after which activated Parkin amplifies ubiquitin signals on outer-membrane substrates and facilitates recognition by the autophagy machinery [13,21,22,23,24,25]. These molecular, organelle-level, and degradative responses operate as a coupled network rather than as a strictly linear cascade: failure at one level increases the burden placed on the others.

### 2.1. Molecular-Level Mqc in PD: Protein Import, Proteostasis and Mitochondrial Renewal

At the molecular level, MQC forms a first line of defense that preserves the mitochondrial proteome and sustains respiratory function through coordinated control of protein import, intramitochondrial proteostasis, and mitochondrial renewal [12,26]. Because most mitochondrial proteins are nuclear-encoded and synthesized in the cytosol, mitochondrial fitness depends on accurate precursor targeting, import, folding, and assembly [27]. Protein import therefore serves as an early stress interface: when it fails, precursors accumulate and proteostatic burden rises before overt organelle loss occurs [27]. This is directly relevant to PD, in which pathogenic α-synuclein can bind translocase of the outer mitochondrial membrane 20 (TOM20) and disrupt mitochondrial protein import [28]. Molecular MQC further relies on chaperones, proteases, and stress-responsive pathways that maintain intramitochondrial proteostasis [12], as well as on a renewal arm of MQC, in which mitochondrial biogenesis and mtDNA homeostasis help preserve respiratory capacity [29,30]. In PD dopaminergic neurons, reduced levels of mitochondrial transcription factor A (TFAM), sirtuin 3 (SIRT3), and other MQC-related proteins support impairment of this molecular maintenance layer [9]. Molecular-level maintenance is therefore closely coupled to organelle-level remodeling, because persistent import, proteostatic, or renewal stress can increase the need for mitochondrial network adaptation.

### 2.2. Organelle-Level Mqc in PD: Mitochondrial Dynamics, Transport, and Local Segregation

At the organelle level, MQC depends on mechanisms that remodel the mitochondrial network and control its positioning. Mitochondrial dynamics comprise fusion, which buffers local defects through content mixing, and fission, which facilitates the segregation of persistently damaged mitochondrial domains from the remaining network [17,18]. Mitochondrial transport and positioning further help determine whether damaged mitochondria remain functionally integrated, become locally confined through transport arrest and spatial segregation, or proceed toward elimination [18,19]. This spatial organization is especially important in neurons, where mitochondria must be continuously moved and retained to match local demands for ATP supply and calcium buffering at axons and synapses [19,20]. In PD, this is particularly consequential: SNpc dopaminergic neurons combine exceptionally large axonal arbors with high basal bioenergetic demand, which likely increases their dependence on efficient mitochondrial transport and network remodeling [31,32]. Organelle-level remodeling is therefore closely linked to selective mitochondrial elimination, because damaged mitochondrial domains often require segregation before efficient mitophagic clearance.

### 2.3. Selective Mitochondrial Elimination in PD: Mitophagy, ER Coupling and Lysosomal Execution

Selective mitochondrial elimination represents another key layer of MQC and is closely coordinated with mitochondrial dynamics, organelle contacts, and lysosomal degradation. Mitophagy removes persistently dysfunctional mitochondria, especially those that have lost their membrane potential, a state termed depolarization [13]. In the best-characterized pathway, PTEN-induced kinase 1 (PINK1) is a serine/threonine kinase that accumulates on the outer mitochondrial membrane when mitochondrial depolarization or impaired import prevents its normal proteolytic processing. Once stabilized there, PINK1 phosphorylates pre-existing ubiquitin and the ubiquitin-like domain of Parkin at Ser65. Phosphorylated ubiquitin promotes Parkin recruitment and primes it for PINK1-dependent activation, enabling activated Parkin to amplify ubiquitin signals on outer mitochondrial membrane substrates [21,22]. However, mitochondrial labeling alone does not complete clearance; effective elimination also requires mitochondrial remodeling, autophagosome formation, and lysosomal degradation [13,23]. ER–mitochondria contact sites provide spatial platforms facilitating mitochondrial constriction, division, and early mitophagy-related processing [16,24]. Mitochondria–lysosome contacts further shape the handling of damaged mitochondria in neurons, and glucosylceramidase beta 1 (GBA1)-mutant dopaminergic neurons show prolonged contacts with impaired untethering and disturbed axonal mitochondrial homeostasis [33]. In PD, this multistep organization is clinically relevant: PINK1 and Parkin (PRKN) implicate defects in damage sensing and commitment to clearance, whereas GBA1 and ATP13A2 indicate that lysosomal execution can also constrain mitochondrial elimination. Taken together, MQC in PD is best understood as a coordinated multilevel system that spans molecular maintenance, organelle-level remodeling, selective elimination, and inter-organelle execution. This framework clarifies why MQC defects may have especially severe consequences in selectively vulnerable neuronal populations.

## 3. Selective Vulnerability as the Entry Point to Mqc-Driven PD Heterogeneity

The multilevel architecture of MQC provides a framework for understanding mitochondrial maintenance, but it does not explain why neurodegeneration in PD is spatially selective and cell-type-selective. This section addresses that problem by placing MQC dysfunction within the biological context of vulnerable neuronal populations. SNpc dopaminergic neurons are not passive targets of mitochondrial injury. Their pacemaking activity, calcium handling, axonal architecture, and dopamine metabolism impose sustained demands on mitochondrial surveillance, repair, redistribution, and turnover. Selective vulnerability therefore provides the cellular setting in which partial MQC defects may become pathologically consequential [9,32].

### 3.1. Selective Neuronal Vulnerability in PD

Selective neuronal vulnerability is a key feature of PD, in which neurodegeneration is distributed unevenly across neuronal populations rather than occurring uniformly throughout the nervous system [32]. The most characteristic pattern is the preferential degeneration of dopaminergic neurons in the SNpc, which contributes directly to the cardinal motor syndrome of PD [1,34]. This pattern is not merely an anatomical observation. Increasing evidence indicates that this vulnerability is rooted in the cell-intrinsic physiological and metabolic properties that impose a chronically elevated stress burden on MQC [35].

SNpc dopaminergic neurons operate under an unusually demanding cellular regime. Their autonomous pacemaking activity drives sustained calcium entry, while their exceptionally large, poorly myelinated axonal arbors and extensive synaptic fields impose further demands; together, these increase the energetic cost of action potential generation, mitochondrial trafficking, axonal transport, and calcium buffering [31,32]. In parallel, dopamine handling and its oxidation can amplify oxidative, mitochondrial, and lysosomal stress [36]. These features narrow the energetic and proteostatic safety margin of nigral dopaminergic neurons and make them especially sensitive to defects in mitochondrial maintenance.

Recent single-cell studies further indicate that vulnerability is heterogeneous even within the SNpc. In human substantia nigra, multiple dopaminergic subpopulations can be identified transcriptionally, but a subset, particularly a ventral-tier population marked by angiotensin II receptor type 1 (AGTR1), shows disproportionate depletion in PD and enrichment for PD genetic risk [37]. This is consistent with earlier neuropathological observations that neuronal loss in the substantia nigra is especially pronounced in the ventral tier [38]. Selective vulnerability in PD should therefore not be understood as a uniform property of all dopaminergic neurons. It is better viewed as a graded and subtype-specific feature shaped by intrinsic stress burden and adaptive capacity.

Importantly, selective vulnerability depends not only on neuron-intrinsic properties but also on regional astrocyte heterogeneity. Ventral midbrain astrocytes display distinct molecular and physiological characteristics, including differences in membrane properties, calcium signaling, oligodendrocyte coupling, and dopamine D2 receptor sensitivity [39]. VTA astrocytes protect both VTA and SN dopaminergic neurons from MPP^+^ toxicity more effectively than SN astrocytes, whereas astrocyte depletion increases VTA neuronal vulnerability. Moreover, VTA astrocytes express substantially higher levels of the neuroprotective factor GDF15 [40]. These findings implicate astrocytes in MQC-related regulation of dopaminergic neuronal vulnerability, although astrocyte-to-neuron mitochondrial transfer in PD remains to be established.

Evidence from non-PD models has suggested that astrocytes can participate in intercellular MQC. Astrocyte-to-neuron transfer of functional mitochondria has been reported after ischemic injury, whereas damaged neuronal mitochondria can be externalized for degradation by adjacent astrocytes [41,42]. However, the extent of intact organelle transfer remains methodologically debated, because transfer of mitochondrial dyes does not necessarily demonstrate transfer of mitochondria. These studies should therefore be viewed as cross-disease precedents rather than evidence for a PD mechanism. Whether bidirectional astrocyte–neuron mitochondrial exchange occurs in the PD-affected midbrain or contributes to differential SN–VTA vulnerability remains unknown.

### 3.2. MQC Dependence as a Mechanistic Basis for Selective Vulnerability

These observations suggest that selective vulnerability can be reframed as unequal dependence on MQC across neuronal populations [10]. SNpc dopaminergic neurons require continuous mitochondrial surveillance, repair, redistribution, and turnover to sustain pacemaking activity, calcium buffering, dopamine handling, and axonal maintenance [43]. Under these conditions, MQC plays a central role in preserving neuronal stability, because mitochondrial fitness must be maintained across molecular, organelle-level, and degradative processes [10].

This dependence reduces the margin for compensation when mitochondrial stress accumulates [9]. Partial defects in mitochondrial protein import, intramitochondrial proteostasis, dynamics, or selective elimination may therefore have greater functional consequences in SNpc dopaminergic neurons than in populations with lower energetic and spatial demands [9,44]. Consistent with this, dopaminergic neurons from PD patients show coordinated reductions in MQC-related proteins, including components of the mitophagy machinery, mitochondrial chaperones, and regulators of mitochondrial protein synthesis and stress adaptation [9].

The graded nature of selective vulnerability further suggests that the compensatory capacity of MQC differs among nigral dopaminergic subtypes [45]. Human single-cell profiling identifies selectively depleted AGTR1-marked dopaminergic neurons in PD [37], and complementary evidence from a non-human primate model is consistent with the existence of vulnerable and relatively resilient nigral dopaminergic phenotypes [46]. Together, these findings suggest that subtype-specific physiological programs and stress-response capacity may influence how much mitochondrial burden a neuron can tolerate before decompensation occurs [45].

This framework offers one way to understand why diverse pathogenic insults may converge on MQC in PD. Genetic defects, α-synuclein pathology, lysosomal insufficiency, oxidative stress, and metabolic aging arise from different upstream contexts, yet each increases the burden placed on mitochondrial maintenance systems. In neurons with high MQC dependence and limited compensatory reserve, these insults are more likely to cross the threshold from adaptive stress response to overt MQC failure. Selective vulnerability therefore helps explain why MQC dysfunction may have especially severe consequences in specific neuronal populations, rather than acting as a uniform pathological process throughout the nervous system.

## 4. Molecular Mechanisms of MQC Dysfunction in PD

**Figure 1:**
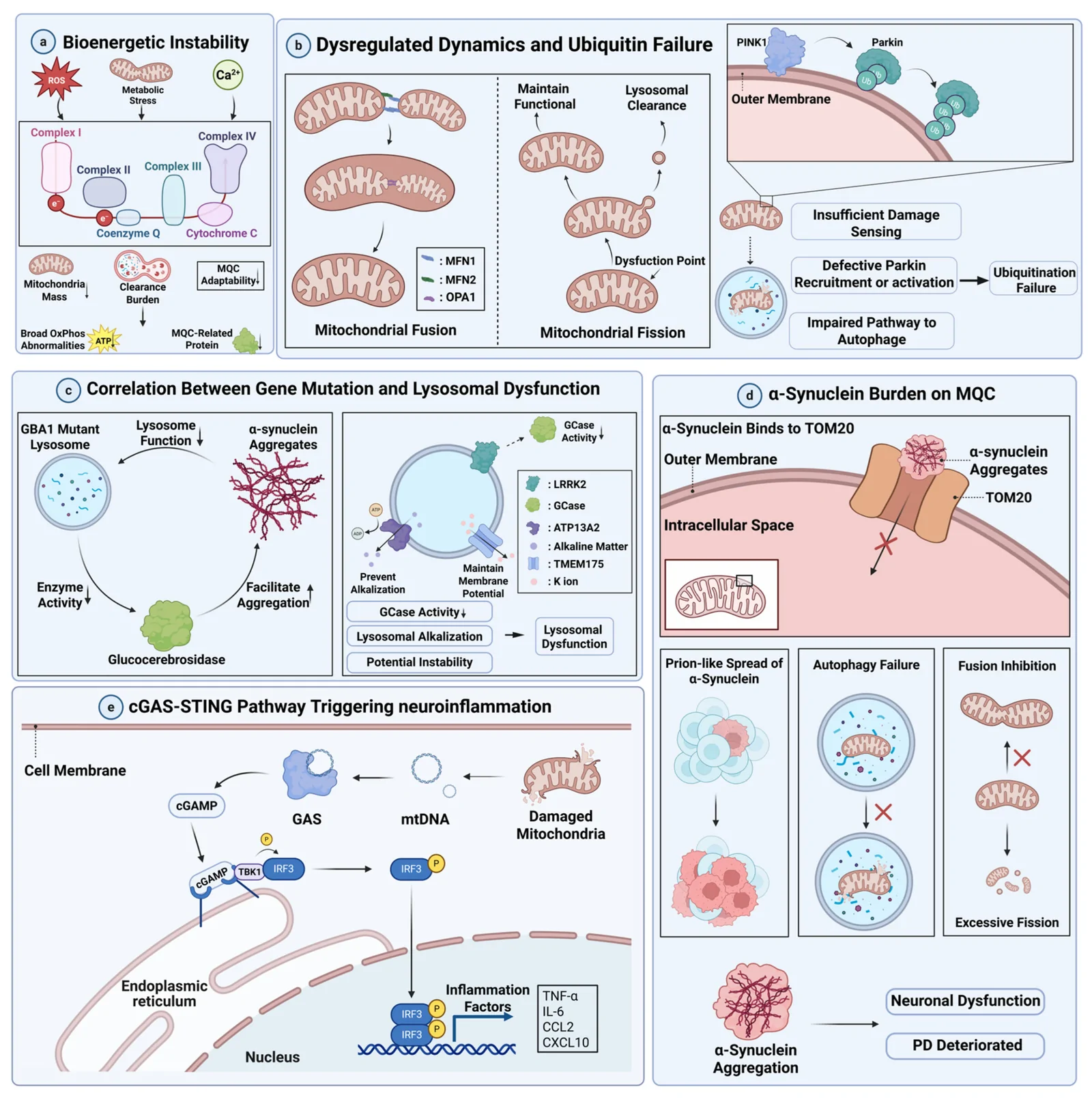
Mechanisms of mitochondrial quality control dysfunction in Parkinson’s disease. Schematic overview of major mechanisms by which impaired MQC contributes to PD pathogenesis. (**a**) Bioenergetic instability arises from oxidative phosphorylation abnormalities, oxidative stress, and calcium burden, increasing mitochondrial damage and overwhelming MQC adaptability despite preserved mitochondrial mass. (**b**) Dysregulated mitochondrial dynamics and defective PINK1/Parkin-dependent ubiquitination impair mitochondrial fusion–fission balance, damage sensing, and mitophagic clearance, resulting in the persistence of dysfunctional mitochondria. (**c**) PD-related genetic factors converge on lysosomal dysfunction, including impaired glucocerebrosidase activity, altered lysosomal acidification, and membrane instability, thereby promoting α-synuclein aggregation and defective degradative capacity. (**d**) α-Synuclein further disrupts MQC by binding TOM20, impairing mitochondrial protein import, inhibiting autophagy and fusion, and favoring excessive fission and neuronal dysfunction. (**e**) Damaged mitochondria can release mitochondrial DNA (mtDNA), which activates the cGAS-STING pathway and downstream TBK1/IRF3 signaling, leading to inflammatory gene expression and neuroinflammation. Together, these interconnected pathways establish a feed-forward cycle of mitochondrial dysfunction, impaired organelle turnover, proteostatic stress, and progressive neurodegeneration in PD. Created in BioRender. Chen, Y. (2026). https://BioRender.com/jnndu2l (accessed on 1 September 2026).

As summarized in Figure 1, MQC dysfunction in PD should not be understood as a linear cascade with a single initiating event. Instead, it is better conceptualized as an interconnected failure network involving bioenergetic instability, impaired mitochondrial damage recognition, defective mitochondrial dynamics, lysosomal–autophagic bottlenecks, α-synuclein-associated proteostatic stress, and inflammatory amplification. These mechanisms are discussed separately below, but they are functionally coupled. A defect in one layer can increase the burden on other MQC modules, especially in neurons with high mitochondrial demand and limited compensatory reserve.

### 4.1. Bioenergetic Instability and Reduced Respiratory Reserve

Mitochondrial dysfunction in PD is often framed in terms of respiratory-chain impairment, but a more relevant concept is bioenergetic instability, in which neurons retain short-term viability yet lose the respiratory reserve required to buffer sustained metabolic, oxidative, and calcium-related stress. This distinction is important for substantia nigra pars compacta dopaminergic neurons because they maintain autonomous pacemaking, extensive axonal arborization, dopamine handling, and local calcium buffering, all of which increase dependence on mitochondrial oxidative phosphorylation [32,47].

Experimental evidence supports the pathogenic relevance of sustained respiratory insufficiency. Selective disruption of mitochondrial complex I in dopaminergic neurons induces progressive parkinsonism, indicating that persistent respiratory-chain impairment can drive PD-relevant phenotypes rather than merely reflect secondary neuronal damage [48]. Human post-mortem evidence further corroborates this view. Dopaminergic neurons from PD midbrain tissue show broad oxidative phosphorylation abnormalities together with reduced abundance of MQC-related proteins, despite preserved mitochondrial mass [9]. These findings suggest that mitochondrial pathology in PD reflects impaired functional adaptability rather than simple loss of mitochondrial content.

Bioenergetic instability also increases the demand placed on downstream MQC mechanisms. When respiratory reserve declines, mitochondria become more vulnerable to depolarization, redox imbalance, and proteostatic stress and damaged components must be repaired, redistributed, segregated, or eliminated to preserve network function. Bioenergetic instability should therefore be viewed both as one major expression of MQC failure and as a stressor that further increases dependence on mitochondrial dynamics, mitophagy, and lysosomal execution [13,49]. When this compensatory capacity is exceeded, persistent mitochondrial damage may do more than reflect MQC failure. It may actively shift vulnerable neurons toward ferroptotic death by sustaining reactive oxygen species (ROS) production, promoting phospholipid peroxidation, and weakening mitochondrial antioxidant defense [50]. In PD, this possibility is supported by evidence that substantia nigra dopaminergic neurons exhibit reduced glutathione peroxidase 4 (GPX4) expression and ferroptosis-related dysregulation of iron-handling genes [51].

### 4.2. Defective Mitophagy and Dysregulated Mitochondrial Dynamics

A central component of MQC is the ability to recognize and eliminate damaged mitochondria. Under basal conditions, PINK1 is imported into healthy mitochondria and degraded after proteolytic processing. When mitochondrial membrane potential is lost or import is impaired, PINK1 accumulates on the outer mitochondrial membrane and phosphorylates ubiquitin and Parkin, thereby activating Parkin-dependent ubiquitin signaling on damaged mitochondria [21,22,25].

However, in PD, failure of ubiquitin-dependent mitochondrial clearance can arise through several partially overlapping mechanisms, including insufficient damage sensing, defective Parkin recruitment or activation, excessive deubiquitination, impaired transmission of ubiquitin signals to the autophagy machinery, and downstream failure of autophagosome–lysosome completion [44,52]. Damaged mitochondria must be recognized, segregated from the functional network, labeled for turnover, and delivered to the autophagy–lysosome system. Impairment at any of these steps can allow dysfunctional mitochondria to persist within neurons, thereby increasing oxidative stress and weakening respiratory resilience [13,53].

PRKN-associated PD illustrates that the link between parkinsonian neurodegeneration and classical Lewy body pathology may be less absolute than traditionally assumed. Bi-allelic PRKN-PD is frequently characterized by a relatively pure nigral degeneration with minimal Lewy body pathology compared with idiopathic PD [54], while contemporary neuropathological frameworks increasingly emphasize that Lewy bodies do not fully define the biological heterogeneity of PD-related disorders [55]. In this sense, Parkin loss of function may be viewed as revealing a partial dissociation between MQC failure, nigrostriatal degeneration, and overt Lewy body formation.

Mitochondrial dynamics are closely coupled with this process. Fusion can dilute local damage through content mixing, whereas fission can facilitate the segregation of damaged mitochondrial domains from the remaining network [17,18]. Parkin-mediated ubiquitination and proteasomal degradation of mitofusins can suppress refusion of damaged mitochondria, thereby helping to isolate mitochondrial fragments marked for clearance [56]. However, if fission occurs without successful mitophagy, fragmented mitochondria may accumulate and contribute to network dysfunction.

This mechanism is particularly relevant to PD because dopaminergic neurons require mitochondrial transport, local positioning, and continuous turnover across long axonal fields. In this context, impaired coordination between fission, clearance initiation, and lysosomal degradation may reduce the ability of neurons to maintain mitochondrial quality over time.

### 4.3. Lysosomal–Autophagic Bottlenecks and Mitochondria–Lysosome Coupling

Mitophagy is not completed by mitochondrial labeling alone. Effective MQC also requires autophagosome maturation, lysosomal fusion, and lysosomal degradation. Lysosomal dysfunction can therefore act as a downstream bottleneck that limits the completion of mitochondrial turnover, even when damaged mitochondria are correctly recognized [13,57]. This point is critical for PD because several genetic and sporadic disease mechanisms converge on lysosomal–autophagic insufficiency.

The GBA1 pathway provides one of the clearest examples. GBA1 encodes glucocerebrosidase (GCase), a lysosomal enzyme required for glycosphingolipid metabolism, and GBA1 mutations are strongly associated with PD risk [58]. Reduced glucocerebrosidase activity can impair lysosomal degradation and promote α-synuclein accumulation. Conversely, α-synuclein accumulation can further disrupt glucocerebrosidase trafficking and lysosomal function, forming a bidirectional pathogenic loop [59]. As illustrated in Figure 1c, this loop links lysosomal insufficiency, α-synuclein proteostasis, and MQC failure rather than treating them as separate pathological processes.

Recent work further shows that lysosomal dysfunction can affect mitochondrial homeostasis through organelle contact sites. In dopaminergic neuronal models carrying GBA1 dysfunction, mitochondria–lysosome contacts are prolonged because of defective contact untethering, leading to disturbed mitochondrial distribution and function [33]. This finding is important because it places lysosomal dysfunction upstream of mitochondrial network abnormalities. It also supports the view that PD-related MQC failure occurs at the interface between organelles, not only within mitochondria themselves.

However, GBA1 is not the only lysosome-related PD gene relevant to this process. Leucine-rich repeat kinase 2 (LRRK2) also intersects strongly with lysosomal–autophagic regulation. LRRK2 has been localized to lysosomal membranes and regulates Rab-dependent membrane trafficking, lysosomal positioning, and lysosomal calcium-related homeostasis, all of which influence downstream degradative function [60]. Importantly, LRRK2 kinase activity has also been shown to regulate neuronal glucocerebrosidase biology, indicating functional convergence between the LRRK2 and GBA1 pathways rather than two isolated genetic programs [61,62]. Consistent with this view, hyperactive LRRK2 signaling can also impair autophagosome transport through Rab dysregulation, thereby disrupting the delivery of degradative cargo in neurons [60].

Additional lysosomal genes further support the idea that ionic and pH homeostasis of lysosomes is critical for PD-related MQC integrity. ATP13A2, a lysosomal ATPase linked to familial PD, contributes to maintenance of lysosomal acidity; PD-associated ATP13A2 dysfunction leads to lysosomal alkalinization and accumulation of α-synuclein, consistent with impaired degradative competence [63]. Similarly, transmembrane protein 175 (TMEM175), a lysosomal ion channel identified as a PD risk factor by genome-wide association studies (GWASs), regulates lysosomal pH balance, and its deficiency has been associated with impaired proteolytic activity and increased α-synuclein aggregation [62,64,65,66]. These findings indicate that lysosomal failure in PD is not limited to defective substrate enzymes such as glucocerebrosidase, but also includes disruption of the ionic environment required for lysosomal function itself.

Taken together, these findings argue that lysosomal–autophagic insufficiency in PD points toward a broader pathogenic principle: when lysosomal degradation, membrane trafficking, or organelle contact regulation is impaired, neurons become less able to complete mitochondrial repair and clearance programs, thereby allowing damaged mitochondria and aggregation-prone proteins to accumulate over time [52,67].

### 4.4. α-Synuclein Pathology as a Modifier of MQC Burden

α-Synuclein pathology is a defining feature of PD, but its relationship with MQC should not be reduced to abnormal aggregation alone. From an MQC perspective, the key question is how α-synuclein accumulation burdens mitochondrial import, intramitochondrial proteostasis, organelle turnover, and lysosomal degradation—a perspective that integrates α-synuclein pathology into the MQC network.

One direct mechanism involves mitochondrial protein import. Misfolded or accumulated α-synuclein can bind TOM20 and inhibit the import of nuclear-encoded mitochondrial proteins, thereby compromising mitochondrial proteome maintenance and respiratory function [28]. Because import failure affects nuclear-encoded proteins broadly, it can impair mitochondrial function before overt organelle loss, connecting α-synuclein proteotoxicity to mitochondrial function before overt organelle loss occurs. This mechanism connects α-synuclein proteotoxicity to mitochondrial maintenance at the molecular level. As noted earlier, α-synuclein can also increase lysosomal and proteostatic burden by forming a bidirectional loop between glucocerebrosidase deficiency and α-synuclein accumulation [59].

α-Synuclein exhibits prion-like propagation: misfolded assemblies act as seeds that recruit soluble endogenous α-synuclein and template its misfolding and aggregation, enabling self-amplification and intercellular spread of pathology. Pathological α-syn can be released from affected cells, taken up by neighboring or anatomically connected cells, and subsequently trigger new rounds of intracellular aggregation, promoting the progression of pathology along neural networks [68]. Moreover, distinct α-synuclein assemblies differ in seeding efficiency, propagation behavior, and neurotoxicity, suggesting that conformational heterogeneity may shape disease phenotypes [69,70]. α-Synuclein aggregation can also hyperactivate dynamin-related protein 1 (DRP1) and promote its accumulation in mitochondria, driving excessive fission. This shortens the length of mitochondria and inhibits fusion, thereby disrupting normal mitochondrial dynamics [71,72]. Furthermore, α-synuclein can also prevent the autophagy of mitochondria and form autophagosomes, but these cannot be cleared [73,74]. The cooperative impact of these α-syn-driven disruptions to fission–fusion balance and mitophagic clearance on MQC failure is illustrated in Figure 1d. As damaged mitochondria and undegraded α-syn species accumulate, they reinforce one another in a vicious cycle that drives MQC failure, neuronal dysfunction, and the progressive worsening of Parkinson’s disease [28,75].

### 4.5. Mitochondrial Stress and Inflammatory Amplification

In response to mitochondrial proteotoxic stress, cells activate the mitochondrial unfolded protein response (${\mathrm{UPR}}^{\mathrm{mt}}$) as an adaptive mechanism to restore protein homeostasis through activating transcription factor 4 (ATF4)-, activating transcription factor 5 (ATF5)-, and C/EBP homologous protein (CHOP)-dependent transcriptional programs [76,77]. However, when mitochondrial damage is persistent and exceeds the buffering capacity of MQC, this compensatory response becomes insufficient, allowing dysfunctional mitochondria to trigger inflammatory cascades. In PD, mitochondrial dysfunction is closely associated with chronic neuroinflammation, including glial activation and increased expression of proinflammatory mediators [78]. In line with this concept, 1-methyl-4-phenyl-1,2,3,6-tetrahydropyridine (MPTP)-treated mice exhibited disturbed mitochondrial homeostasis, elevated ${\mathrm{UPR}}^{\mathrm{mt}}$-related proteins, increased levels of IL-1β, IL-6, TNF, and NLR family pyrin domain containing 3 (NLRP3), and enhanced ionized calcium-binding adapter molecule 1(IBA1)-, glial fibrillary acidic protein (GFAP)-, and transmembrane protein 119 (Tmem119)-positive gliosis [79].

Under these conditions, MQC failure can also convert mitochondrial stress into inflammatory signaling. The cyclic GMP-AMP synthase (cGAS)–stimulator of interferon genes (STING) pathway is an important signaling axis that enables the body to sense cytoplasmic DNA and trigger the innate immune response [80]. Mitochondrial damage can lead to the release of mtDNA into the cytosol, where it acts as a danger-associated molecular pattern and activates cGAS, thereby triggering STING signaling. Activation of the cGAS-STING pathway in microglia and other responsive cell types promotes the production of inflammatory mediators, such as tumor necrosis factor (TNF)-α, IL-6, C-C motif chemokine ligand 2 (CCL2), and C-X-C motif chemokine ligand 10 (CXCL10), as illustrated schematically in Figure 1e, which in turn amplifies neuroinflammation [81,82].

Overall, the mechanisms discussed in this section indicate that MQC dysfunction in PD is distributed across several interconnected layers: reduced bioenergetic reserve, impaired mitochondrial damage sensing, dysregulated dynamics, lysosomal–autophagic bottlenecks, α-synuclein-associated import and proteostatic stress, and inflammatory amplification. The relevant genes and phenotypes related to hereditary Parkinson’s disease are summarized in Table A1. The next section examines how different etiological entries may weight these mechanisms differently, thereby producing distinct MQC failure configurations that contribute to PD heterogeneity.

## 5. Heterogeneity in PD: MQC as an Explanatory Framework

PD heterogeneity is expressed across multiple levels, including etiological background, anatomical distribution of pathology, dominant symptom profile, and rate of disease progression. The preceding sections have shown that despite their different entry points, both familial and sporadic forms of PD may involve partially convergent disturbances in MQC, although the specific affected modules, causal position, and clinical relevance are likely to differ across genetic backgrounds, ages at onset, and disease stages. The question, therefore, is not simply whether MQC is involved in PD, but how disturbances within the same mitochondrial maintenance network may be translated into distinct clinical trajectories. In this context, MQC provides a useful framework for linking etiological diversity to subtype formation, system-specific vulnerability, and between-patient differences in progression.

### 5.1. Initial MQC Differences in sPD and fPD

PD is commonly divided into fPD and sPD according to etiological context [34,83]. Familial PD refers to cases with familial aggregation or a pathogenic genetic contribution, including monogenic forms that account for a minority of PD cases [2,34,83]. Sporadic PD represents the majority of patients and usually lacks a clear Mendelian cause [34,83]. Its risk is shaped by aging, common genetic susceptibility, environmental exposure, and cellular stressors that act over time [2,83]. This distinction is useful for defining disease entry points, although it does not fully explain downstream biological heterogeneity.

In an MQC-centered framework, fPD is informative because it reveals how defined pathogenic lesions can bias specific parts of the mitochondrial maintenance network [8,34,84]. These genes have been addressed above. Sporadic PD is different in that it more often reflects cumulative pressure on MQC rather than disruption of a single inherited node [34,83]. Aging reduces proteostatic, autophagic, lysosomal, and mitochondrial reserve [85], while dopamine metabolism, α-synuclein accumulation, environmental exposure, and polygenic risk may further increase mitochondrial stress [34,83]. Therefore, some forms of sporadic PD may involve a gradual accumulation of stress across multiple MQC-related processes, in which several quality control layers become progressively insufficient under sustained burden [34,85].

Both fPD and sPD represent etiologically heterogeneous routes into PD biology, but both can converge on MQC as a central disease hub [8,9,84]. In fPD, pathogenic variants provide more traceable molecular entries that weight particular MQC modules, whereas sPD susceptibility can progressively erode MQC reserve. This convergence supports the use of MQC as an organizing framework for PD heterogeneity [8,44,84].

### 5.2. MQC Related to Abnormalities May Contribute to Differential Neuronal Vulnerability and Clinical Heterogeneity

MQC imbalance may contribute to PD heterogeneity by both increasing neuronal vulnerability and shaping patterns of selective vulnerability across neural systems, which may in turn influence the order and extent of clinical involvement [8,9,34,84]. Within this interpretive framework, clinical subtypes such as mild motor-predominant, intermediate, and diffuse malignant PD may reflect uneven system involvement and differential compensatory capacity. MQC-related abnormalities could plausibly contribute to these processes, although they should not be regarded as a discrete basis for defining these clinical subtypes. This prospect remains compatible with the previously described subtype structure and reported prognostic stratification [86,87].

A clinical profile consistent with the mild motor-predominant subtype may be associated with a pattern of MQC impairment that appears largely confined to the nigrostriatal circuit, although direct evidence for spatially restricted MQC dysfunction in human PD remains limited [84,86]. This interpretation is biologically tenable, given that substantia nigra dopaminergic neurons exhibit a pronounced reliance on mitochondrial integrity owing to their autonomous pacemaking activity, calcium-handling burden, and highly elaborate axonal arborization [32,44,47].

By contrast, when dysfunction emerges earlier in the lower brainstem, olfactory structures, or autonomic networks, the phenotype may more closely resemble body-first or non-motor-predominant PD [88]. In such cases, constipation, hyposmia, rapid eye movement (REM) sleep behavior disorder (RBD), and autonomic dysfunction are frequently observed as prodromal manifestations, often preceding overt parkinsonism by several years [89,90]. When MQC impairment is broader and less effectively compensated, pathology is more likely to extend beyond the nigrostriatal and brainstem systems into limbic and cortical networks—a pattern more consistent with the diffuse malignant subtype and its greater non-motor burden and faster functional deterioration [9,34,86,87].

Accordingly, the difference among mild motor-predominant, intermediate, and diffuse malignant PD may be interpreted in part as reflecting variation in the extent and distribution of network vulnerability, to which MQC dysfunction could plausibly contribute [9,86,87]. Overall, PD subtypes may be viewed, at least in part, as clinical manifestations of differential vulnerability and compensatory capacity across neural systems, within which MQC reserve represents one plausible determinant of when and where dysfunction becomes clinically apparent [9,34,84,85].

### 5.3. Interindividual Differences in MQC Reserve, the Breadth of Pathway Failure, and Aging May Further Amplify PD Heterogeneity

The clinical consequences of MQC dysfunction are unlikely to be explained by its mere presence alone, because PD neurons exhibit coordinated changes across multiple MQC proteins rather than a uniform abnormality [9]. A more plausible interpretive framework is that PD heterogeneity may arise in part from between-patient differences in mitochondrial compensatory capacity, in whether MQC abnormality remains localized or becomes more broadly coordinated across pathways, and in the degree to which aging lowers the threshold for decompensation. This interpretation is consistent with previous work on the MQC framework and on aging-related decline in mitochondrial adaptability [44,91]. These factors may help explain why patients exposed to broadly similar pathogenic pressures nevertheless develop markedly different phenotypes and rates of progression [34,87].

First, the capacity of vulnerable neurons to maintain mitochondrial homeostasis may vary between patients, particularly under conditions of aging, cumulative damage, impaired turnover, and declining bioenergetic resilience [91]. Variations in mitophagy, mitochondrial proteostasis, antioxidant buffering, and mitochondrial biogenesis may influence how long vulnerable neuronal populations can tolerate metabolic and oxidative stress before functional decompensation becomes clinically relevant, because these processes operate as interconnected components in the MQC system rather than as isolated pathways [9,44,49].

Second, PD heterogeneity may also be shaped by whether MQC disruption remains relatively limited to a single process or extends across several interconnected layers of the system, because mitochondrial homeostasis depends on coordinated mechanisms including protein folding and degradation, mitochondrial dynamics, vesicular trafficking, and mitophagy [44,49]. When impairment is largely confined to one process, partial compensation by parallel MQC mechanisms may still be possible. By contrast, simultaneous defects in mitochondrial proteostasis, protein import, organelle turnover, redox buffering, and lysosomal clearance would be expected to reduce this compensatory capacity and produce broader, less reversible dysfunction, consistent with the coordinated MQC abnormalities observed in PD neurons [9]. Severe phenotypes may therefore arise not simply from the magnitude of an isolated lesion, but from failure of MQC as an integrated network, an interpretation that is clinically compatible with the diffuse malignant end of the subtype spectrum [86,87].

Aging is likely to further amplify this divergence by weakening the cellular systems that normally buffer mitochondrial stress and proteostatic burden [91,92]. With advancing age, the accumulation of mitochondrial DNA damage, oxidative stress, impaired metabolic signaling, and declining lysosomal and autophagic capacity progressively reduces cellular adaptability, thereby lowering the threshold at which mitochondrial dysfunction becomes clinically consequential [91]. PD heterogeneity may therefore reflect not only the anatomical distribution of early vulnerability, but also the amount of residual compensatory capacity, the number of MQC layers affected, and the extent to which aging has eroded the ability of neural systems to withstand stress [9,34].

### 5.4. MQC as an Integrative Framework for PD Heterogeneity

PD heterogeneity is better understood not as a secondary clinical epiphenomenon, but as a core biological feature of the disease. Within an MQC-centered framework, differences in anatomical vulnerability, pathway-specific versus network-wide dysfunction, residual compensatory capacity, and the impact of aging may together contribute to distinct disease trajectories. The major dimensions of this heterogeneity and their potential MQC-based interpretations are summarized in Table A2. MQC should therefore be considered not only a mechanism of neuronal injury, but also a useful conceptual framework for interpreting why PD may manifest and progresses so variably across patients.

## 6. Potential Translational Implications of an MQC Framework

MQC-related biology may help organize therapeutic hypotheses across several PD-relevant pathways, but current evidence does not establish MQC as a discrete patient-stratification axis. The most clinically developed examples involve the GBA–lysosome and LRRK2 pathways, whereas direct modulation of mitophagy remains at an early stage. Their translational evidence should be considered at three separate levels: biological enrichment of a study population, pathway-proximal target engagement, and demonstrated clinical benefit. Accordingly, this section uses MQC as a research framework for evaluating mechanistically linked interventions and evidence gaps, rather than as a validated system for assigning individual patients to treatment [93,94].

### 6.1. Candidate MQC-Related Readouts and Their Current Limitations

Disease-modifying therapy remains an unmet need in PD, and biological classification frameworks are increasingly being used to define research populations. SynNeurGe classifies PD using evidence of pathological α-synuclein, neurodegeneration, and pathogenic genetic variants, and is explicitly proposed as a research framework that requires prospective validation [94]. These biological anchors can support trial enrollment or subgroup analysis, but they are not MQC biomarkers. MQC is therefore not proposed here as a fourth SynNeurGe axis or as an alternative clinical classification system.

At present, no clinical-grade biomarker measures MQC as an integrated multilevel network, identifies the proposed MQC failure configurations in individual patients, or supports treatment assignment. Existing measurements instead capture selected components or adjacent pathways. Phosphorylated ubiquitin at Ser65 (pS65-Ub) is a candidate pathway-proximal readout of PINK1–Parkin signaling and mitochondrial damage, but current biofluid findings require replication before clinical use [95]. GCase activity and glycosphingolipid levels can inform the GBA–lysosome pathway, whereas phosphorylated LRRK2, phosphorylated Rab10, and urinary bis(monoacylglycerol) phosphate can indicate LRRK2 target or pathway engagement [96,97,98]. These measures do not capture MQC as a whole and have not been validated as prognostic markers or surrogate endpoints for clinical benefit. More general measures, including serum FGF21, GDF15, and blood mitochondrial DNA copy number, have also shown insufficient or context-dependent discrimination in PD [42]. The present biomarker discussion is therefore limited to candidate research and pharmacodynamic readouts rather than validated MQC stratification biomarkers [99].

### 6.2. Clinically Advanced MQC-Linked Routes: GBA–Lysosome and LRRK2 Pathways

The GBA–lysosome axis is one of the most clinically evaluated MQC-linked therapeutic routes. GBA1-associated PD directly implicates lysosomal degradation, which contributes to α-synuclein clearance and the terminal execution of autophagic and mitophagic turnover [58,59]. Ambroxol, a pharmacological chaperone of glucocerebrosidase, is being tested in the ongoing phase 3 ASPro-PD trial, which includes prospective analysis according to GBA1 status [100]. In a randomized phase 2 trial on Parkinson disease dementia, ambroxol was generally well tolerated and showed central exposure and GCase-related target engagement, but no benefit was demonstrated on the primary or secondary cognitive outcomes [101]. These studies support the biological plausibility of lysosomal rescue and the feasibility of genotype-aware evaluation, but they do not establish clinical efficacy or validate MQC-guided treatment selection.

Other GBA-directed approaches further illustrate both the promise and limitations of pathway-specific therapy. LY3884961, an intracisternal AAV9-GBA1 gene therapy, has entered first-in-human phase 1/2a evaluation in patients with GBA1-associated PD, indicating that direct restoration of glucocerebrosidase activity is now clinically testable [102]. By contrast, substrate reduction with the glucosylceramide synthase inhibitor venglustat did not show a beneficial treatment effect in the phase 2 MOVES-PD trial, despite a clear mechanistic rationale and acceptable safety [103]. Together, these studies show that the GBA field is biologically coherent and clinically active, but they also demonstrate that correcting a pathway-proximal biochemical abnormality may be insufficient to modify disease course.

A second clinically important route is centered on LRRK2. Pathogenic LRRK2 activity is linked to Rab GTPase phosphorylation, vesicular trafficking, lysosomal regulation, and immune-related cellular functions, placing LRRK2 within the trafficking and lysosomal environment in which MQC is executed [104,105]. BIIB122/DNL151 is a brain-penetrant LRRK2 kinase inhibitor that showed pharmacokinetic and pharmacodynamic evidence of target engagement in early-phase studies, including reductions in LRRK2 pathway biomarkers [96]. Its clinical development has subsequently been revised: the phase 3 LIGHTHOUSE study in LRRK2-associated PD was discontinued because of trial complexity and timeline considerations, while the LUMA and BEACON studies continue to evaluate BIIB122 in early-stage PD and LRRK2-associated PD using efficacy and pharmacodynamic endpoints [60,106]. This trajectory highlights both the feasibility of mechanism-based intervention and the practical difficulty of testing disease modification in genetically defined PD.

Transcript-lowering strategies provide another LRRK2-directed approach. BIIB094/ION859 is an intrathecal antisense oligonucleotide designed to reduce LRRK2 mRNA. A first-in-human phase 1 study evaluated its safety, tolerability, pharmacokinetics, and pharmacodynamics in patients with PD, supporting the feasibility of LRRK2-lowering approaches while leaving clinical efficacy unresolved [107]. These programs provide opportunities to test pathway-defined intervention and pharmacodynamic monitoring while clinical efficacy remains unresolved.

### 6.3. Direct Mitophagy Enhancement: Conceptually Precise but Clinically Early

Direct mitophagy enhancement is conceptually the closest strategy to repairing MQC itself, but it remains earlier in clinical development than GBA- or LRRK2-directed approaches. PINK1 activation aims to strengthen the mitochondrial damage-sensing axis, whereas USP30 inhibition aims to enhance ubiquitin-dependent mitophagy by reducing deubiquitination at damaged mitochondria [108,109]. These approaches directly target the machinery that determines whether damaged mitochondria are marked, retained in the quality control pathway, and cleared.

Direct mitophagy modulation has entered early human testing, but clinical development remains limited to safety, pharmacokinetic, and proof-of-mechanism evaluation. ABBV-1088, a PINK1-activating compound, has been studied in phase 1 healthy-volunteer protocols. One single-ascending-dose study was completed without publicly posted results, whereas a multiple-ascending-dose study was terminated for strategic reasons [110,111]. MTX325, a USP30 inhibitor, is being evaluated in a multi-part phase 1 study in which multiple dosing in patients with PD is ongoing [112]. No clinical efficacy data currently establish that direct PINK1 activation or USP30 inhibition modifies PD progression, and no validated biomarker has been shown to identify patients who are likely to benefit.

### 6.4. Barriers to MQC-Linked Disease Modification

The first major barrier is biological heterogeneity. Broad enrollment based only on clinical diagnosis may dilute a therapeutic signal when participants do not share the biology targeted by an intervention [93,94,99]. Genetic enrichment is currently most defensible for GBA1- or LRRK2-directed approaches because the relevant pathway is implicated by genotype. By contrast, no validated biomarker currently identifies a subgroup whose dominant abnormality is impaired mitophagy or another integrated MQC configuration. This limitation is particularly important in late-onset idiopathic PD, in which MQC abnormalities may be downstream of, or shared with, α-synuclein pathology, aging, inflammation, and established neurodegeneration rather than representing a primary and specific disease driver. MQC-related measurements in these populations should therefore remain exploratory unless their relationship to treatment response is prospectively demonstrated.

The second barrier is the gap between target engagement and clinical efficacy. Venglustat produced the expected glycosphingolipid pharmacodynamic effect but did not improve clinical outcomes in GBA1-associated PD [103]. Ambroxol showed central exposure and GCase-related target engagement in Parkinson disease dementia, but cognitive benefit was not demonstrated [101]. Similarly, BIIB122 markedly inhibited LRRK2 pathway biomarkers in LUMA without slowing clinical progression [113]. These findings show that pathway-proximal readouts can confirm biological activity of an intervention but cannot currently be treated as validated surrogate endpoints for disease modification.

The third barrier is timing. PD-related biological abnormalities may begin before motor diagnosis, whereas many trials enroll patients after substantial network dysfunction has already developed [99,114]. If MQC failure has already spread from a dominant entry point to broader network collapse, single-node intervention may have limited clinical effect. This is especially important because MQC is not a linear pathway. It includes mitochondrial import, proteostasis, dynamics, mitophagy, lysosomal degradation, and inter-organelle coordination. Once several layers fail together, restoring one node may not be sufficient to recover neuronal resilience.

The fourth barrier is endpoint selection. Conventional clinical scales may be insensitive to modest slowing of disease progression over short trial durations, especially when symptomatic variability and compensatory mechanisms obscure disease-modifying effects [99]. MQC-linked trials therefore require endpoints that can connect target engagement to downstream biological effects and long-term clinical trajectories. This requires combining clinical outcomes with biomarkers of pathway engagement, neurodegeneration, and disease progression.

### 6.5. Requirements for Evaluating MQC-Linked Therapeutic Hypotheses

Future trials should not assign patients to treatment on the basis of an unvalidated MQC configuration. A defensible sequence is to define the study population using established biological anchors, select a pathway-proximal pharmacodynamic marker matched to the intervention, and prospectively test whether biomarker modulation predicts a clinically meaningful outcome. Genetic enrichment may be appropriate for GBA1- or LRRK2-directed interventions. For late-onset idiopathic PD and direct mitophagy interventions, candidate MQC-related readouts should initially be used for exploratory mechanistic analysis rather than treatment assignment.

Established biological markers can provide complementary context for such studies. α-Synuclein seed amplification assays can identify synucleinopathy biology, whereas genetic, imaging, and neurodegeneration markers can further characterize a trial population [93,94,115]. Multimodal initiatives such as STRAT-PARK illustrate the broader movement toward data-driven biological subtyping beyond clinical diagnosis alone [116]. These approaches may reduce heterogeneity in trial enrollment, but they should not be described as validating an MQC subgroup or an MQC-based treatment-selection algorithm.

Pathway-proximal pharmacodynamic markers should be matched to the intervention being tested. In LRRK2-directed trials, pS935-LRRK2, pRab10, and urinary bis(monoacylglycerol) phosphate can support assessment of target or pathway engagement [96,98]. In GBA-directed trials, glucocerebrosidase activity and glycosphingolipid substrate measures provide pathway-relevant readouts [97,103]. These measures are pathway-specific rather than MQC-wide, and their relationship to long-term clinical benefit remains unvalidated. Direct mitophagy trials will require analytically validated measures of mitochondrial damage sensing, mitophagy flux, lysosomal completion, and downstream neuronal injury before any prognostic or treatment-predictive use can be considered.

Overall, MQC-based translation should not be framed as a single universal therapy for all PD. Rather, its value lies in organizing mechanistically matched interventions for biologically defined subgroups, as summarized in Table A3. The most realistic path forward is not simply to enhance mitochondrial function in all patients, but to identify which MQC failure configuration dominates in a given subgroup, confirm target engagement with pathway-proximal biomarkers, and test whether this engagement modifies disease-relevant trajectories over time.

### 6.6. Limitations and Open Questions

Several limitations should be acknowledged. First, this article is an interpretive synthesis rather than a systematic review; we did not apply predefined search or inclusion criteria, so the selection of supporting studies may be subject to bias. Second, much of the human evidence for coordinated MQC-protein loss derives from a limited number of primary datasets—most notably single-neuron and single-nucleus studies—and findings obtained in cellular and animal models may not fully generalize to human PD. Third, the proposed links between specific MQC modules and clinical subtypes, anatomical spread, and progression rate are largely inferential: direct evidence for spatially patterned MQC dysfunction in human PD remains limited, and a causal relationship has not been established. Fourth, MQC overlaps with other explanatory frameworks—including proteostatic, neuroinflammatory, and prion-like mechanisms—and is emphasized here for integrative clarity rather than as an exclusive cause of PD. Fifth, the translational section summarizes early-phase and ongoing programs, some referenced from trial registries or press releases, whose conclusions may change as data mature; demonstrated target engagement does not establish disease modification. Accordingly, the framework presented here should be regarded as hypothesis-generating and will require prospective, biomarker-anchored validation.

## 7. Conclusions

PD is increasingly understood as a biologically and clinically heterogeneous disorder rather than a single uniform entity. This heterogeneity is expressed at multiple levels, including etiological background, dominant pathogenic mechanisms, symptom onset sequence, and progression rate. Although familial PD and sporadic PD arise from partially distinct origins, they may converge on a shared downstream state of MQC failure. In this review, MQC is considered a multilevel and functionally coupled network that includes mitochondrial proteostasis, redox balance, dynamics, biogenesis, and selective elimination of damaged mitochondria. Because neurons—particularly nigrostriatal dopaminergic neurons—are highly dependent on sustained energy production, calcium buffering, and long-distance organelle transport, they are especially vulnerable to even modest disruption of mitochondrial maintenance.

From this perspective, MQC dysfunction provides a mechanistic framework for linking diverse pathogenic insults to selective neuronal degeneration and phenotypic diversity in PD. Distinct initiating factors, including PD-related mutations, α-synuclein pathology, lysosomal–autophagic defects, and age-associated metabolic stress, may affect different components of the MQC system, yet ultimately produce convergent consequences such as respiratory instability, oxidative stress, impaired mitochondrial turnover, and defective organelle coordination. This convergence may help explain why patients show marked variation in clinical presentation while still sharing core pathological features. Moreover, regional differences in mitochondrial stress and compensatory reserve may influence which neural systems fail first, thereby shaping symptom sequence and progression rate. Overall, MQC should not be regarded as the sole cause of PD, but as an organizing principle that integrates molecular pathology, selective vulnerability, and disease heterogeneity, with important implications for mechanistic stratification and targeted therapeutic development.

## Data Availability

No new data were created or analyzed in this study. Data sharing is not applicable to this article.

## Abbreviations

AAV9	adeno-associated virus 9
AGTR1	angiotensin II receptor type 1
ATF4	activating transcription factor 4
ATF5	activating transcription factor 5
ATP	adenosine triphosphate
ATP13A2	ATPase cation transporting 13A2
cGAS	cyclic GMP-AMP synthase
CCL2	C-C motif chemokine ligand 2
CHOP	C/EBP homologous protein
CXCL10	C-X-C motif chemokine ligand 10
DRP1	dynamin-related protein 1
ER	endoplasmic reticulum
fPD	familial Parkinson’s disease
GBA1	glucosylceramidase beta 1
GCase	glucocerebrosidase
GFAP	glial fibrillary acidic protein
GPX4	glutathione peroxidase 4
GWAS	genome-wide association study
IBA1	ionized calcium-binding adapter molecule 1
IL-1β	interleukin-1 beta
IL-6	interleukin-6
LRRK2	leucine-rich repeat kinase 2
MPTP	1-methyl-4-phenyl-1,2,3,6-tetrahydropyridine
MQC	mitochondrial quality control
mRNA	messenger RNA
mtDNA	mitochondrial DNA
NLRP3	NLR family pyrin domain containing 3
PD	Parkinson’s disease
PINK1	PTEN-induced kinase 1
PRKN	parkin RBR E3 ubiquitin protein ligase
Rab	Ras-related in brain
ROS	reactive oxygen species
SIRT3	sirtuin 3
SNpc	substantia nigra pars compacta
sPD	sporadic Parkinson’s disease
STING	stimulator of interferon genes
TFAM	mitochondrial transcription factor A
TMEM119	transmembrane protein 119
TMEM175	transmembrane protein 175
TNF	tumor necrosis factor
TNF-α	tumor necrosis factor alpha
TOM20	translocase of the outer mitochondrial membrane 20
UPRmt	mitochondrial unfolded protein response
USP30	ubiquitin specific peptidase 30

## Appendix A

**Table A1:** Hereditary Parkinson’s disease-associated genes and reported MQC phenotypes.

Gene	Primary Cellular Function	Reported MQC Phenotypes in PD-Associated Mutation Contexts	References
*PINK1*	Mitochondrial kinase, mitophagy initiation	Impaired damage sensing; abolished Parkin phosphorylation/recruitment; accumulated depolarized mitochondria	[21,22,25]
*PRKN*	E3 ubiquitin ligase, downstream effector of PINK1	Defective damaged mitochondria labeling; impaired mitofusin degradation; minimal Lewy body pathology despite nigral degeneration	[54,56]
*GBA1*	Lysosomal glucocerebrosidase, glycosphingolipid metabolism	Reduced lysosomal degradative activity; prolonged mitochondria–lysosome contacts; bidirectional α-synuclein/MQC failure loop	[33,58,59]
*LRRK2*	Lysosome-associated kinase, Rab-dependent membrane trafficking regulation	Disrupted lysosomal position/homeostasis; impaired autophagosome transport; GBA1 pathway functional convergence	[60,61,62]
*ATP13A2*	Lysosomal ATPase, regulator of lysosomal acidity	Lysosomal alkalinization; loss of proteolytic competence; secondary α-synuclein-triggered MQC bottlenecks	[63]
*TMEM175*	Lysosomal ion channel, pH balance maintenance	Impaired lysosomal proteolysis; increased α-synuclein aggregation; disrupted ionic environment for mitochondrial turnover	[62,64,65,66]
*SNCA*	Presynaptic protein, aggregate-prone modulator of intracellular trafficking	TOM20-mediated mitochondrial import inhibition; excessive DRP1-dependent fission; blocked late-stage mitophagy clearance	[28,68,71,72,73,74]

Note: This table summarizes the main MQC-related phenotypes of PD-linked genes, involving mitophagy, mitochondrial dynamics, and lysosomal coupling. Effects vary by variant; *GBA1* and *TMEM175* are PD risk factors, not strictly Mendelian hereditary causes.

**Table A2:** PD heterogeneity in an MQC-centered framework.

Heterogeneity Dimension	PD Manifestation	Potential MQC-Based Interpretation	Biological Significance	References
Etiological heterogeneity	PD includes familial and sporadic forms with distinct etiological backgrounds.	Different etiological entries may converge on MQC through either discrete genetic defects or cumulative stress burden.	Distinct disease origins may still share a common mitochondrial pathogenic hub.	[8,9,34,44,83,84,85]
Mechanistic heterogeneity	The relative contribution of α-synuclein pathology, lysosomal dysfunction, mitochondrial damage, and oxidative stress varies across patients.	MQC may serve as a convergence node linking multiple pathogenic pathways.	Mechanistic diversity may reflect different modes of perturbing the same mitochondrial maintenance system.	[8,9,34,44,49,85]
Heterogeneity in symptom onset sequence	Motor and non-motor symptoms do not emerge in a uniform order across patients.	Regionally uneven MQC failure may shape system-specific vulnerability and symptom sequence.	Symptom order may reflect differences in early anatomical involvement.	[9,34,84,88]
Heterogeneity in progression rate	Patients differ markedly in the speed of clinical deterioration and multisystem involvement.	Differences in MQC reserve and compensatory capacity may influence the rate of decompensation.	Progression rate may depend on the extent of integrated MQC failure.	[9,84,85,86,87]

Note: Key dimensions of PD heterogeneity may partly converge on disruption of the MQC network. MQC is presented here as an integrative framework rather than a determinant of any single clinical subtype.

**Table A3:** MQC-based stratification framework and representative therapeutic routes in PD.

Therapeutic Route	MQC-Linked Rationale	Representative Agents/Programs	Current Translational Status	References
GBA–lysosome axis	Restores lysosomal clearance linked to α-synuclein and organelle turnover	Ambroxol; LY3884961; venglustat	Ambroxol in phase 3; LY3884961 in phase 1/2a; venglustat negative in phase 2	[58,59,103]
LRRK2 pathway	Modulates Rab signaling, trafficking, and lysosomal regulation	BIIB122/DNL151; BIIB094/ION859	BIIB122 in ongoing studies; LIGHTHOUSE discontinued; BIIB094 in phase 1	[96,104,105,107]
Direct mitophagy enhancement	Enhances mitochondrial damage sensing or mitophagy flux	ABBV-1088; MTX325	Early clinical stage; proof-of-mechanism; no efficacy evidence yet	[13,108,109]
Biomarker-guided stratification	Matches therapy to genetic and pathway-defined biology	SynNeurGe; α-synuclein seed amplification; STRAT-PARK	Trial-enrichment framework; not routine clinical practice	[93,94,116]

Note: This table summarizes representative MQC-linked therapeutic and stratification approaches in PD. “Translational status” indicates the current stage of development and evidence for target/pathway engagement, not confirmed disease-modifying efficacy.
